# Identification of primary and secondary metabolites and transcriptome profile of soybean tissues during different stages of hypoxia

**DOI:** 10.1016/j.dib.2018.09.122

**Published:** 2018-10-15

**Authors:** Isabel Duarte Coutinho, Liliane Marcia Mert Henning, Silas Aurelian Döpp, Alexandre Nepomuceno, Larissa Alexandra Cardoso Moraes, Juliana Marcolino-Gomes, Christian Richter, Harald Schwalbe, Luiz Alberto Colnago

**Affiliations:** a Embrapa Instrumentação, Rua XV de Novembro, 1452, São Carlos 13560-970, São Paulo, Brazil; b Embrapa Soja, Rodovia Carlos João Strass, Distrito de Warta, Londrina 86001-970, Paraná, Brazil; c Center for Biomolecular Magnetic Resonance, Johann Wolfgang Goethe-University Frankfurt, Max-von Laue-Str. 7, Frankfurt/M. 60438 Germany

**Keywords:** Soybean, Nuclear magnetic resonance, Primary metabolites, Secondary metabolites

## Abstract

NMR and chromatography methods combined with mass spectrometry are the most important analytical techniques employed for plant metabolomics screening. Metabolomic analysis integrated to transcriptome screening add an important extra dimension to the information flow from DNA to RNA to protein. The most useful NMR experiment in metabolomics analysis is the proton spectra due the high receptivity of ^1^H and important structural information, through proton–proton scalar coupling. Routinely, databases have been used in identification of primary metabolites, however, there is currently no comparable data for identification of secondary metabolites, mainly, due to signal overlap in normal ^1^H NMR spectra and natural variation of plant. Related to spectra overlap, alternatively, better resolution can be find using ^1^H pure shift and 2D NMR pulse sequence in complex samples due to spreading the resonances in a second dimension. Thus, in data brief we provide a catalogue of metabolites and expression levels of genes identified in soy leaves and roots under flooding stress.

**Specifications table**

**Table:** 

Subject area	*chemistry, biology, agronomy*
More specific subject area	*Metabolomic screening*
Type of data	*Table, figure*
How data was acquired	*NMR and RNAseq*
Data format	*Analyzed*
Experimental factors	*1D and 2D NMR experiments were used for the metabolite annotation. The LC-DAD-MS was used to support NMR data. Statistical analysis tool such as principal component analysis and variance analysis were performed for physiological and grown parameters and metabolite relative concentration. The expression levels of genes in response to flooding stress was obtained.*
Experimental features	*The metabolites were assigned from chemical shift and coupling constant data and compared with literature information. The complete assignment was confirmed by 2D NMR information. The retention time and molecular mass data from LC-DAD-MS was helpful for accurate metabolite annotation. The expression of the genes in response to different hypoxia levels was assessed by analysing an RNA-seq library database derived from soybean leaves under flooding stress.*
Data source location	*Londrina/Brazil and Frankfurt/Germany*
Data accessibility	*Data is available with this article.*
Related research article	*Coutinho ID, Henning, LMM, Döpp SA, Nepomuceno A, Moraes AC, Marcolino-Gomes J, Richter C, Schwalbe H, Colnago LA. Flooded soybean metabolomic analysis reveals importante primary and secondary metabolites involved in the hypoxia stress response and tolerance. Environ. Exp. Bot. 2018 153:176–187.*

**Value of data**

• The metabolite annotation is useful to be combined with untargeted and target metabolomic approach and might be contributed to a data bank of chemical shift and retention time of primary and secondary metabolites in soybean hydroalcoolic extracts.
• Determine the main metabolic pathway is affected by flooding stress.
• The expression of genes related to the key enzymes involved in the sucrose degradation and alanine and GABA metabolism contribute to explain the metabolic alterations observed in under flooding.
• The resume of variance analysis is important to understand the statistical analysis results.

## 1 Data

Detailed description of metabolite identification in soybean leaves and roots extract, multivariate analysis of secondary metabolites identified in soybean tissues, expression of genes and statistical analysis.

## 2 Experimental design, materials and methods

### 2.1 Sample preparation for metabolomic analysis

The extracts were obtained according related research article [1], [2].

### 2.2 Instrumentation

#### 2.2.1 NMR analysis

The spectra were acquired at a temperature of 298 K on a Avance 600 spectrometer operating at 600, 1699 MHz using a 5 mm Prodigy TCI probe. The ^1^H pure shift experiment was performed by reset_psyche_1d.pr NMR pulse sequence for homonuclear broadband decoupling [3], [4]. The spectra were acquired with a 4.50 s presaturation delay and acquisition time of 3.64 s (64k points). The chirp pulse were generated in the shape tool of topspin with length of pulse 15 ms, total sweep-width 10 kHz, size of shape 10,000 and smoothed in 20%. The gradient pulse aligned with the centre of two chirp pulse was range to 1.0–2.0%. The spectra windows in F1 and F2 were set to 80 and 5 kHz, respectively. The number of t1 (number of chunks) was set to 32–128. The pure shift interferogram was constructed using a script processing provide by Bruker. The ^1^H 1D NMR experiments were performed according to related research article [1]. Phasing and baseline correction were carried out within the instrument software.

#### 2.2.2 LC-MS/MS system

LC-MS/MS system was used to support ^1^H NMR data. The soybean genotypes BR4 and E45 under control conditions were analysed by LC-DAD-MS using LC-DAD-ESI system consisting of a Shimadzu 20 A HPLC equipped with a LC-20AD quaternary pump, a SPD-M20A photodiode array detector, a SIL-20A thermostated autosampler and a CTO-20A column compartment, coupled to a Bruker Ion Trap, with a heated ESI source. UV spectra were acquired from 230 to 400 nm. Mass spectra were acquired in negative and positive modes over *m/z* range of 100–1000, in separated runs. Operating parameters were as follow: source voltage, 4.5 kV, sheath gas, 9.00 L/min dry gas, 40 psi nebulizer and dry temperature, 300 °C. Automatic MS-MS was performed on the three most abundant ions of each scan. An isolation width of *m/z* 3 was used and precursors were fragmented by CID with normalized collision energy of 60. The data analyses were performed using Data Analysis software. The chromatographic runs were performed using Kinetex® C-18 column (1.9 µm, 30 × 2.1 mm i.d., Phenomenex), which was maintained at 25 °C. The gradient of elution was performed with water/0.1% formic acid (A) and acetonitrile/0.1% formic acid (B) under the following conditions: 0 min, 5% B; 30 min, 40%B; 35 min, 100%B; 40 min, 100%B. Flow rate at 1.0 mL/min and injection volume of 1 µL.

### 2.3 Data analysis

The ^1^H NMR data ranging from 6.00 to 8.50 ppm were converted to ASCII files using Bruker TopSpin 3.5. The data preprocessing and Principal Component Analysis (PCA) from ^1^H NMR were performed using MATLAB R2016b and PLS-Toolbox. The data analysis was performed according related research article [1].

### 2.4 Gene expression analysis

RNAseq libraries of soybean roots under hypoxic stress, obtained by Nakayama et al. [5] and were used in this study. The experimental design consisted of two soybean cultivars (BR4 and E45) submitted to different stress durations: 0.5 h, 4 h, and 28 h [1].

### 2.5 Statistical analysis

Data from physiological parameters, biomass accumulation, and metabolomic analysis showed a normal distribution and were submitted to the analysis of variance [1].

## 3 Data analysis

### 3.1 Metabolite identification

The metabolites identified were valine (1) δ 0.99 of 6H (d, 7.0), ethanol (2) δ 1.19 of 3H (t, 7.6), lactate (3) δ 1.33 (d, 1.33), hydroxybutyrate (4) δ 1.34 (s), alanine (5) δ 1.48 of 3H (d, 7.3), GABA (6) δ 1.88 3H (q, 7.2), δ 2.30 of 2H (t, 7.5), δ 2.99 of 2H (t, 7.5), acetate (7) δ 1.89 of 3H (s), glutamic acid (8) δ 2.05 of 3H (m), δ 2.14 of 3H (m), δ 2.35 (m), asparagine (12) δ 2.86 (dd, 7.9, 16.6), lysine δ 3.03 of 3H (t, 7.5) and glycine (15) δ 3.57 (s) of 2H. Malic acid (9) succinic acid (12), citric acid (13), choline (15), pinitol (17), myo-inositol (18), fumarate (27) and formic acid (39) were assigned using the signals at 2.36 (q, 9.6), 2.38 (s), 2.38 (s), 2.53 (d, 16.6), 3.19 (s), 3.59 (s), 3.60 (t, 9.4), 6.49 (s) and 8.44 (s) ppm, respectively. β-glucose (19) and α-glucose (21) were identified using the characteristic signals of the anomeric protons at 4.61 (d, 7.91) and 5.20 (d, 3.9). Fructose (18) was assigned using the signal at δ 3.99 (dd, 2.7, 10.0), sucrose (23) at δ 5.39 (d, 4.0) and trehalose (20) at δ 5.19 (d, 4.6).

The signal from δ 6 to 10 were attributed to phenylpropanoids and identified four hydroxycinnamic acids, four flavonols and three isoflavones, which had the structure confirmed by 1D and 2D NMR, LC-DAD-MS/MS experiments. The metabolites *trans*-3-caffeoylquinic acid (24), *cis*-3-caffeoylquinic (23), *trans*-coumaroylquinic acid (26) and *cis*-coumaroylquinic acid (25) were identified in mixture based on differences intensity of H7′/6′. The characteristic signals of hydroxycinnamic derivatives were detected as pairs of doublets at δ 6.06/7.07 and 6.54/7.81, corresponding to *cis-p*-coumaric acid and *trans-p*-coumaric acid, δ 6.51/7.74 and 5.93/7.16 corresponding to *trans-*caffeoyl and *cis-*caffeoyl acid (31), due to coupling of the olefinic protons with Z (*trans*, *J *= 16.0 Hz) and E (*cis*, *J* = 12.0 Hz) configurations (Figure SM3). The COSY and J-Resolved experiments were useful for confirming the presence of signals from a pair olefinic hydrogens from compound 26 that overlapped with the intense signal of fumaric acid (δ 6.53 s). The presence of hydroxycinnamic acid derivatives was supported by UV due to a characteristic absorbance at 300–330 nm corresponding to cinnamoyl systems and confirmed by LC-MS/MS analysis. The compounds 23–26 come out at retention time 4.8, 5.5, 6.4 and 7.2 min. *Trans/cis*-caffeoylquinic acid and *trans*/*cis*-coumaroylquinic acid had precursor ion *m/z* 371 [M-H-18]^−^ and *m/z* 355 [M-H-18]^−^. The MS/MS spectra of isomers showed a base peak product ion of *m/z* 191 [quinate-H]^−^.

Five kaempferol glucosides were identified, three of which are kaempferol triglucosides and two diglucosides. Kaempferol derivatives showed 2 peaks at δ 6.56 (3H, d, *J* = 1.8 Hz) and 6.34 ppm (3H, d, *J* = 1.8 Hz) consistent with the meta protons H-6 and H-8 on A-ring and an AA′BB′ system at 8.07/7.99 (6H, d, 8.6 Hz, H-2′,6′) and 7.00 (6H, d, 8.8 Hz, H-3′,6′) corresponding to the protons on B-ring of aglycone.

Homonuclear scalar couplings corresponding to phenolic acids and kaempferol derivatives were collapsed in singlet lines and significantly improved resolution in aromatic region, allowing the assignment of three kaempferol isomers (28, 29, 30) due to presence of three singlet lines at 8.0 ppm corresponding to J_A′X′_ system (Fig. 4). The phenolic compounds occur in low concentration in plants and aromatic region spectra showed the cost of sensitivity using pure shift method, but maintains advantage of obtaining simplified singlet resonances. Therefore, the better resolution of pure shift methods reveals the potential of PSYCHE 1D as deconvolution tool.

Kaempferol-3-*O-*α-rhamnosyl-di-β-glucoside isomer I (28) was identified as major compound, the sugar moiety shows two overlapped proton signals at 4.72 (1H, d, *J* = 7.5) corresponding to two anomeric proton of a *β*-glucosyl (H-1″/1‴) and a methyl signal 1.12 (3 H, d, *J* = 6.2 Hz) in the high-field region was assigned to rhamnose and 4.39 (1 H, d, *J* = 1.0) were assignable to the H-1 of an *α*-rhamnosyl proton. In the ^1^H and ^13^C NMR values for all the carbons were assigned on the basis of HSQC and are given in Table 1. The placement of the sugar unit was established at C-3 position on the basis UV spectra *λ*_max_ of 265–345 nm indicative that hydroxyl at C-3 is not free. In addition, the structure was further supported by of key HMBC correlations C-4 and H1″. The LC-MS/MS analysis was employed to support NMR data and the Kaempferol-3-*O-*α-rhaminosyl-di-β-glucoside isomer showed [M+H]^+^ peak at *m/z* 757 eluted at 14 min. The MS/MS spectra showed ions *m/z* 611, 595 and 287 in positive mode. The fragment ion *m/z* 611 corresponds to cleavage of glycoside bond (loss of 146 Da – indicative of an arabinosyl moiety), *m/z* 595 corresponds loss of 162 Da indicative of a glucosyl moiety and *m/z* 287 is relative to aglycone Kaempferol.

**Table 1:** Chemical shifts (δ) and coupling constants (Hz) of the primary metabolites identified in hydroalcoholic extracts of soybean roots and leaves.

**Metabolite**	**δ**^**1**^**H (multiplicity, J Hz)**	^**13**^**C (HSQC)**
Valine (1)	0.99 (d, 7.0), 2.27 (m)	–
Ethanol (2)	1.33 (d, 6.6)	
Lactate (3)	1.33 (d, 6.6)	–
Hydroxyisobutyrate (4)	1.34 (s)	23.9
Alanine (5)	1.46 (d, 7.4)	17.9
GABA (6)	1.88 (q, 7.2), 2.30 (t, 7.4), 2.99 (t, 7.5)	24.9, 35.7
Acetate (7)	1.89 (s)	–
Glutamic acid (8)	2.05 (m), 2.14 (m)	29.7
Malic acid (9)	2.36 (q, 9.6, 15.3), 2.66 (dd, 15.3, 3.5)	43.6
Succinic acid (10)	2.38 (s)	33.0
Citric acid (11)	2.53 (d, 16.6)	47.1
Asparagine (12)	2.86 (dd, 7.9, 16.6), 2.96 (dd, 4.2, 12.2)	–
Lysine (13)	3.03 (t, 7.5)	–
Choline (14)	3.19 (s)	–
Glycine (15)	3.57 (s)	–
Pinitol (16)	3.59 (s)	61.1
Myo-inositol (17)	3.60 (t, 9.4), 4.11 (sbr)	–
Fructose (18)	4.19 (d, 3.6)	100.8
β-Glucose (19)	4.61 (d, 7.9)	97.4
Trehalose (20)	5.19 (d, 4.6)	76.1
α-Glucose (21)	5.20 (d, 3.9)	93.5
Sucrose (22)	5.39 (d, 4.0), 4.19 (d, 8.8)	92.1
*cis*-caffeoylquinic acid (23)^L^	5.90 (H8, d, 12.4), 6.72 (H2, d, 8.2), 6.96 (H6, dd, 8.2, 2.0), 7.03 (H5, d, 2.0), 7.16 (H7, d, 12.2).^a^	–
*trans-*caffeoylquinic acid (24)^L^	6.49 (H8, d, 16.0), 6.71 (H2, d, 8.2), 6.99 (H6, dd, 8.2, 2.0), 7.07 (H5, d, 2.0), 7.80 (H7, d, 16.0).^a^	–
*cis*-*p*-coumaroylquinic acid (25)^L^	6.07 (H8, d, 12.3), 7.09 (H7, d, 12.6), 6.86 (H3′5′, d, 8.4), 7.59 (H2′, 6′, d, 8.3), 5.42 (H1″, d, 1.3).^a^	117.5 (C8), 146.7 (C7), 116.4 (C3′,C5′), 133.2 (C2′, C6′), 74.7 (C1″)
*trans*-*p*- coumaroylquinic acid (26)^L^	6.42 (H8, d, 16.0), 7.75 (H7, d, 16.0), 6.92 (H3′5′, d, 8.4), 7.59 (H2′, 6′, d, 8.3).^a^	115.6 (C8), 147.7 (C7), 115.2 (C3′,C5′), 131.9 (C2′, C6′), 74.6 (C1″)
Fumarate (27)	6.49 (s)	136.8
Kaempferol-3*-O-α-*rhamnosyl-di- *β*-glucoside isomer I (28)^L^	6.56 (H6, d, 1.8), 6.34 (H8, d, 1.9), 7.00 (H3′5′, d, 8.6), 8.07 (H2′6′, d, 8.8), 4.72 (Glu-H1″/Glu-H1‴,d, 7.5), 4.39 (Rha-H1⁗, d, 1.0), 1.11 (Rha-H6‴, d, 6.2).^a^	96.6 (C8), 101.9 (C6), 131.5 (C2′,6′), 115.7 (C3′,6′), 99.6 (C1″), 100.7 (C1‴), 16.5 (C6‴), 79.3 (C5″).
Kaempferol-3*-O-α-*rhamnosyl-di- *β-*glucoside isomer II (29)^L^	6.56 (H6, d, 1.8), 6.34 (H8, d, 1.9), 7.03 (H3′5′, d, 8.6), 8.02 (H2′6′, d, 8.8), 4.98 (Glu-H1″, d, 7.5), 4.77(Glu-H1‴, d, 7.5), 4.53 (Rha-H1⁗, d, 1.0), 1.03 (H6‴, d, 6.2), 3.81 (H5″, m).^a^	96.6 (C8), 101.9 (C6), 131.5 (C2′, 6′), 115.7 (C3′, 6′), 100.2 (C1″), 100.8 (C1‴), 16.5 (C6‴), 79.3 (C5″).
Kaempferol-3-*O- α -*di-rhaminosyl-*β-*glucoside (30)^L^	6.56 (H6, d, 1.8), 6.34 (H8, d, 1.9), 7.03 (H3′5′, d, 8.6), 8.04 (H2′6′, d, 8.8). a	
Kaempferol-*O-α-*rhaminosyl-*β-*glucoside (31)^L^	6.56 (H6, d, 1.8), 6.34 (H8, d, 1.9), 7.03 (H3′5′, d, 8.6), 8.02 (H2′6′, d, 8.8), 5.45 (Glu-H1″, d, 7.5), 4.51 (Rha-H1⁗, d, 1.0).^a^	–
Daidzein (32)^R^	6.83 (H8, d, 1.9), 6.93 (H6, dd, 2.2, 8.8), 7.80 (H5, d, 8.8), 6.99 (H3′, 5′, d, 8.5), 7.41 (H2′, 6′, d, 8.5), 8.13 (H2, s). a	–
Daidzin (33)^R^	7.31 (H8, d, 1.9), 7.24 (H6, dd, 2.2, 8.9), 7.80 (H5, d, 8.9), 6.97 (H3′, 5′, d, 8.5), 7.40 (H2′, 6′, d, 8.5), 8.13 (H2, s), 5.25 (Glu-H1″, d, 7.2). a	101.0 (C8), 115.7 (C6), 127.7 (C5), 154.5 (C2), 115.5 (C3′,5′), 130 (C2′,6′)
Malonyldaidzin (34)^R^	7.30 (H8, d, 2.0), 7.25 (H6, dd, 2.2, 8.2), 6.97 (H3′, 5′, d, 8.5), 7.37 (H2′,6′, d, 8.5), 8.14 (H2, s)	
Formic acid (35)	8.44 (s)	–
Trigoneline (36)^L^	9.12 (s)	–

a Supported by LC-MS/MS data. L: detected only in leaf extract. R: detected only in root extract. The underlined signals were used to obtain the relative metabolite concentrations.

Kaempferol-3-*O-*α-rhamnosyl-di-β-glucoside isomer II (29) presented the same signal patterns of compound 28, except to downfield shift of the H-2′,6′ proton (8.02 ppm), as well as high field shift of the corresponding β-glucoside anomeric proton at 4.98 (1 H, d, 7.4 Hz) and 4.77 (1 H, d, 7.7 Hz), with respect to the Kaempferol-3-*O-*α-rhaminosyl-di-β-glucoside I. The C-5‴ signal (79.3) of glucose showed a downfield shift of 2.3 ppm in comparison with the corresponding C-5‴ signal (77.1) of isomer I, indicating the difference of both kaempferol triglucosides is due to conformation of hydroxyl at C5″ position. Kaempferol triglucoside isomer II showed [M+H]^+^ peak at *m/z* 757 eluted at 14.3 min. The MS/MS spectra showed similar fragment ions *m/z* 595 and 287 in positive mode compare to isomer I. The fragment ion *m/z* 595 corresponds to cleavage of glycoside bond (loss of 146 Da – indicative of an glucosyl moiety) and *m/z* 287 is relative to aglycone Kaempferol.

Kaempferol-3-*O-α-*di-rhaminosyl-*β-*glucoside (30) presented the same signal patterns of compounds 28 and 29, except to downfield shift of the H-2′,6′ proton (8.04 ppm). The presence of compound x was supported by LC-MS/MS data from peak at 15 min with a pseudomolecular ion *m/z* 741 [M-H]^−^. The MS/MS spectrum (negative mode) showed ions *m/z* 595 and 444 related to cleavage of the two rhamnosyl units and *m/z* 287 corresponding to cleavage of the glucosyl linkage.

Kaempferol-3-*O-α-*rhamnoside-*β-*glucoside (**31**) showed the same aglycone signal patterns of compound 28 in aromatic region, one anomeric proton signals at 5.45 (1 H, d, *J* = 3.73) was assignable to H-1 of a *α*-glucosyl proton and 4.51 corresponding to rhaminosyl moiety. The compound 31 was identified in with retention time 16.5 min in the total ion chromatogram with a pseudomolecular ion *m/z* 595 [M+H]^+^. The MS/MS spectrum showed ions *m/z* 433 and 287 related to cleavage of the rhamnosyl and glucosyl linkage, respectively.

Daidzein (32), daidzin (33) and malonyldaidzin (34) were identified as major isoflavones from extract of soybean roots (Figure SM2). These isoflavones are common in soybean tissues and the Table 1 shows complete assignment based on literature [8].

**Fig. 1:**
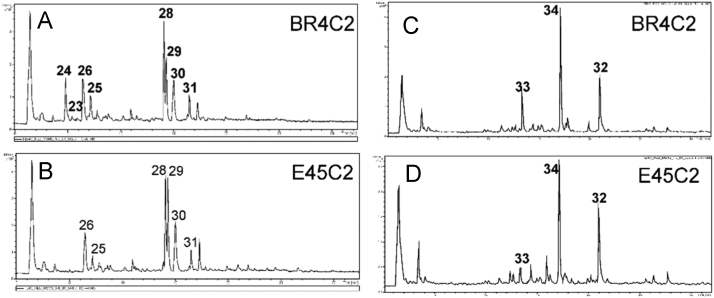
Total Ion Chromatogram (TIC) of hydro alcoholic extract of soybean leaves (A, B) and roots (C, D).

The fluctuation of isoflavones concentration in ^1^H-NMR spectrum of Br4C genotypes was fundamental to spectral assignment due to intensity differences of H-2 and H-5 in a clearly downfield region. The aglycone Daidzein was identified as major and showed 3 signals at δ 6.83 (1 H, d, *J* = 1.9 Hz), 6.93 (1 H, dd, *J* = 2.2, 8.8 Hz) and 7.98 (1 H, d, *J* = 8.8 Hz) characteristic with trisubstituted system on A-ring, 2 signals corresponding to AA′BB′ system at δ 6.97 (2 H, d, 8.1 Hz) and 7.37 (2 H, d, 8.1 Hz) and signal δ 8.13 corresponding to H-2, latter characteristic of isoflavone. The glucosides isoflavones Daidzin and malonyldaidzin presented the same signal patterns of Daidzein corresponding to AA′BB′ system on B-ring. Daidzin showed characteristic signals corresponding to trisubstituted system on A-ring at δ 7.24 (1 H, dd, *J* = 2.2, 8.9 Hz, H-6) and 7.31 ppm (1 H, d, *J* = 2.2 Hz, H-8). The sugar moiety showed signal at 5.25 ppm (1 H, d, 7.2 Hz) and was assignable to H-1 of a *β*-glucosyl proton. Malonyldaidzin shows the same signals patterns of Daidzin, except to downfield shift of H-8 at 7.30 (1 H, d, 2.8 Hz) and H-6 at 7.25 ppm (1 H, dd, 2.2 and 8.2 Hz). The major isoflavones identified by ^1^H-NMR were confirmed using LC-DAD-MS/MS data. Daidzin, malonyldaidzin and daidzein were eluted at 13.4, 17.0 and 21.0 min. This was corroborated by the sequence of elution from C-18, compounds glycosides elute before than aglycone. Daidzin was identified had precursor ion *m/z* 417 [M+H]^+^. MS/MS of *m/z* 417 showed a peak at *m/z* 255 corresponding to aglycone daidzein. Malonyldaidzin showed [M-H]^−^ peak at *m/z* 501 and a base peak product ion of *m/z* 253 [M-248]^−^ corresponding to aglycone daidzein (Fig. 1).

## 4 Multivariate analysis of secondary metabolites identified in hydroalcoolic extracts of soybean tissues

**Fig. 2:**
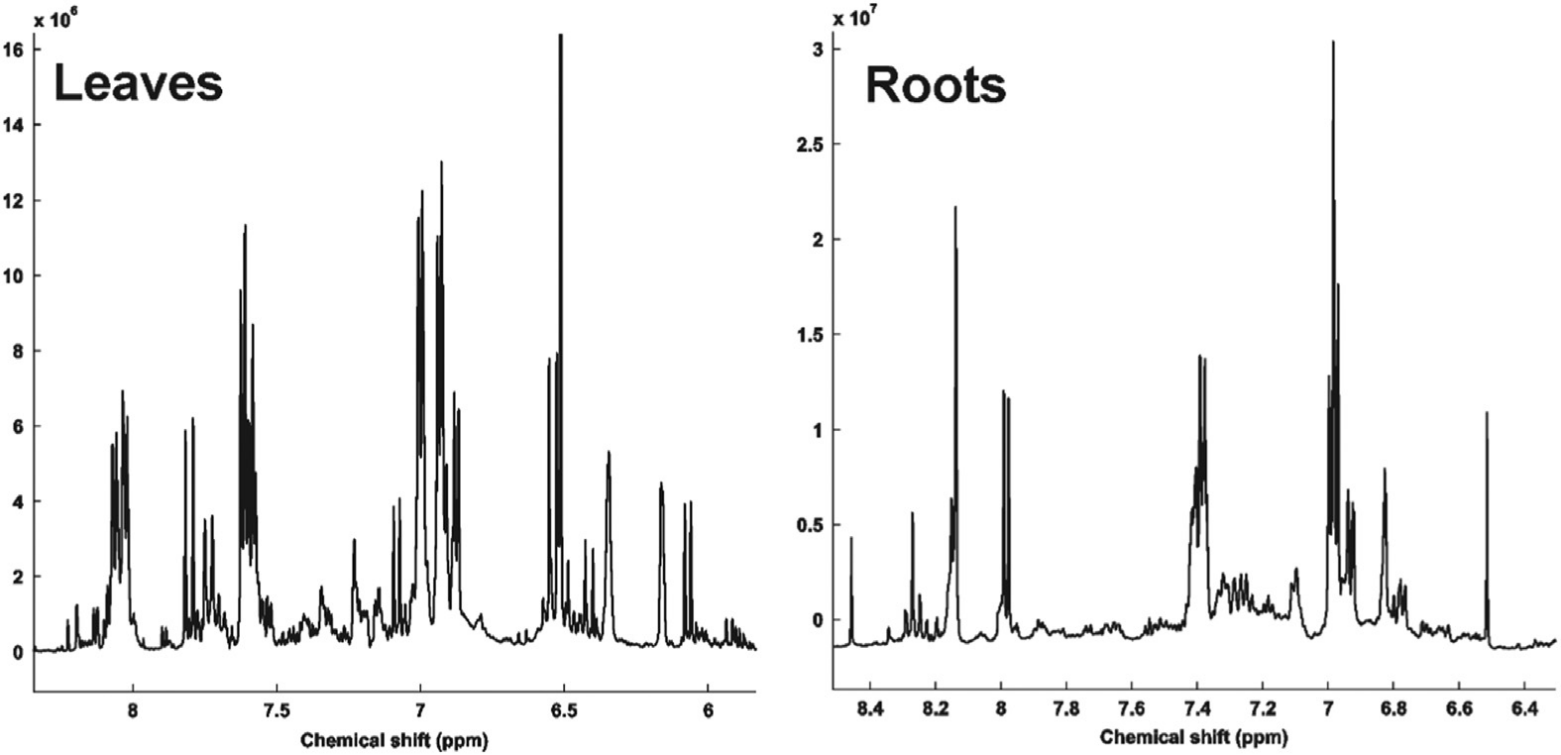
Mean ^1^H NMR spectra region 6.00–8.50 ppm corresponding to main secondary metabolites identified in soybean extract and selected for PCA.

**Fig. 3:**
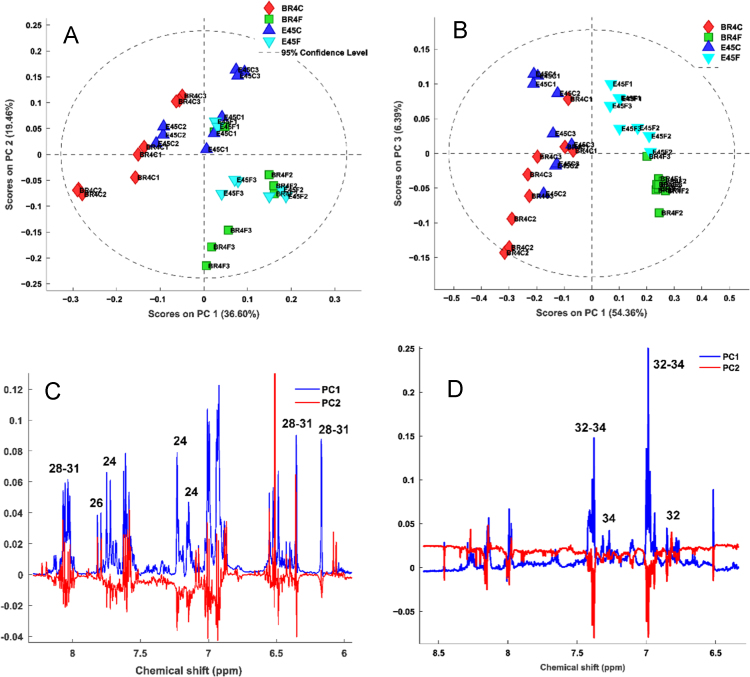
Principal Component Analysis based on ^1^H NMR spectra of soybean tissues. (A) Scores plot performed from ^1^H NMR spectra (δ 6.00–8.50) of hydroalcoholic extract of soybean leaves and roots (B). (C) PC1 *versus* PC3 loadings plot performed from ^1^H NMR spectra (δ 6.00–8.50) of hydroalcoholic extract of soybean leaves and roots (D).

**Fig. 4:**
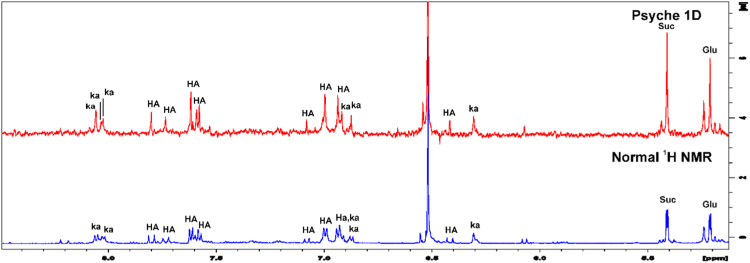
^1^H and 1D PSYCHE spectrum of alcoholic soybean leaves extract in D_2_O/MEOD (8:2 v/v). The region between 5.00 and 8.50 ppm corresponding to chemical shift of phenolic compounds identified. HA (hydroxycinnamic acids) and Kaempferol (Ka) derivatives.

See Fig. 2, Fig. 3, Fig. 4 and Table 2.

**Table 2:** Resume of variance analysis performed for physiological and grown parameters: Stomatal conductance (Gs), Photosynthesis (A), Chlorophyll (CH), Roots dry matter (RDM), Shoot dry matter (SDM) and Total plant dry matter (TDM). (SV) Source of Variation; (DF) Degrees of freedom.

**S.V**	**D.F**	Mean square (MS)					
		**Gs**	**A**	**Chlorophyll**	**RDM**	**SDM**	**TDM**
G	1	0.06615*	44.9634*	0.000041*	0.481667*	0.357704	1.669538*
WC	1	0.176817*	477.6660*	0.000386*	0.260417*	3.658204*	5.870704*
G*WC	1	0.01215	0.110704	0.000005	0.016017	0.175104	0.297037
Error	8	0.003603	5.708316	0.000006	0.014932	0.069369	0.127891
CV		30.65	23.08	26.51	16.78	19.92	17.44
Mean		0.195833	10.35042	0.009425	0.728333	1.322083	2.050417

* *P* value <0.05.

## 5 Expression levels of genes

The expression ratio (fold-change, fc) of genes was performed by dividing transcript abundance values (in RPM = Reads per Mapped Million) from plants under hypoxic and normoxic conditions. The statistical significance of DEGs were obtained by using Bioconductor package edgeR [6], corrected by Benjamini and Hochberg method [7]. We only considered as DEGs those showing fold-change _ 2 (up), _ -2 (down), adj. p-value _ 0.01, and with more than 20 mapped reads (RPM _ 9) in at least one of the two compared libraries. See Table 3.

**Table 3:** Expression levels of genes involved in sucrose degradation and alanine and GABA metabolism. Data obtained from RNAseq libraries of soybean roots under hypoxic stress. Cultivars BR4 and E45 – Times of stress (0.5 h, 4 h, and 28 h). The respective genes were analyzed comparing the hypoxia stress treatment to the control, at each time‐point, and generating fold change values. Red/blue means up/down regulated respectively.

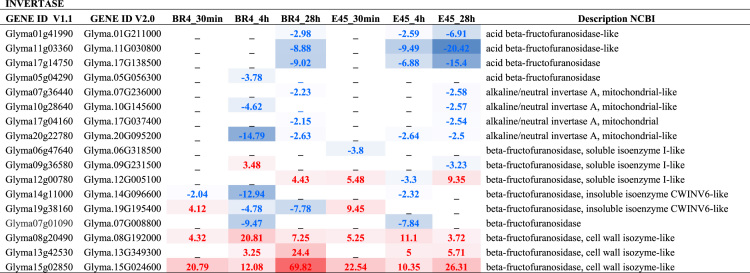
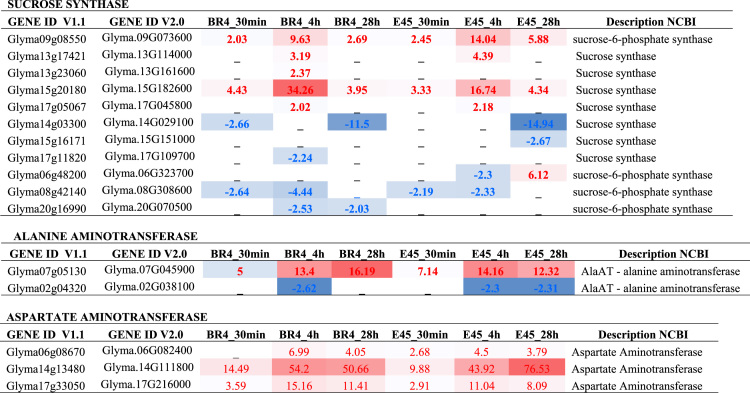
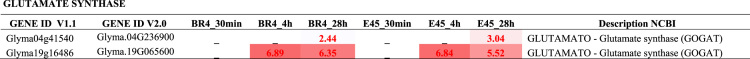

## 6 Statistical analysis

See Table 4, Table 5.

**Table 4:** Resume of variance analysis for metabolites identified in leaves. (SV) Source of Variation; (DF) Degrees of freedom. (DAF) Days after flooding.

S.V	D.F	**Carbohidrates**			**Organic acids**					**Amino acids**				
		Sucrose	Fructose	Glucose	Acetate	Citrate	Succinate	Fumarate	Malate	Alanine	Gaba	Choline	Pinitol	Trigonelline
**Mean square (MS) 2 DAF**														
G	1	0.001055	0.180983	1.111364	0.000036	0.007159	0.00013	0.041513	1.90045*	0.000686*	0.000101	0.006134*	0.041196	0.000011
WC	1	0.002349	0.017457	0.054392	0.000151	0.539455	0.000295	0.155952*	11.04442*	0.001385*	0.011495*	0.001636	0.010878	0.000041
G*WC	1	0.003337	0.178535	0.025089	0.000016	0.00907	0.000845	0.001629	0.628056	0.000268*	0.001523	0.000559	0.787508	0.000295
Error	8	0.002615	0.052895	2.498878	0.000055	0.005275	0.000196	0.008096	0.149417	0.000032	0.000392	0.000662	0.109505*	0.000075
CV		20.96	11.95	15.9	10.39	13.67	12.93	17.22	13.31	10.04	13.01	11.8	13.44	12.61
Mean		0.0862417	1.924775	3.5157917	0.0715833	0.5313583	0.108225	0.5226	2.9046	0.0561083	0.15205	0.2180417	2.4617083	0.068575
	
**Mean square (MS) 7 DAF**														
G	1	0.03596*	1.123571*	0.134747	0.000006	0.196634*	0.0048*	0.015783*	0.291383	0.005768*	0.000577	0.002241	0.209881	0.001372*
WC	1	0.00071	1.673355*	3.84495*	0.000837*	1.586969*	0.00122	0.167986*	17.853188	0.04619*	0.007272	0.023444*	1.176254*	0.000635*
G*WC	1	0.000589	0.86559*	2.16682*	0.000012	0.495036*	0.003523	0.038715*	0.847434	0.006389*	0.000144	0.001801	1.083723*	0.000008
Error	8	0.001097	0.021838*	0.040782	0.000042	0.004671	0.000785	0.002896	0.360963	0.000041	0.000241	0.000437	0.043628	0.000055
CV		0.046032	9.77	7.76	9.47	9.6	18.79	11.34	20.65	5.93	16.08	9.28	8.92	11.44
Mean		23.19	1.5128083	2.5371167	0.0681833	0.7121	0.1490833	0.47465	2.9100767	0.1082917	0.0966167	0.22545	2.3420833	0.0650083
	
**Mean square (MS) 12 DAF**														
G	1	0.002239*	2.011955*	0.144256*	0.000766*	0.063948*	0.000249*	0.012078	0.700592*	0.000888*	0.002688*	0.009269*	4.113606*	1.20E-07
WC	1	0.007346*	10.360208*	37.628438*	0.001848*	0.035317*	0.000328*	0.152258*	0.07904*	0.018236*	0.042721*	0.099791*	7.98293*	0.000131*
G*WC	1	0.005577*	0.0823669	0.782903*	0.000448*	0.029284*	0.000073	0.007455	0.405132*	0.000372*	0.002766*	0.003616*	0.807097*	0.000484*
Error	8	0.000535	0.060403	0.025487	0.000025	0.001166	0.000021	0.00363	0.007464	0.000035	0.000175*	0.001298	0.027105	0.000006
CV		18.34	10.62	4.17	6.31	9.04	5.44	11.2	2.71	6.16	8.62	4.31	4.91	3.46
Mean		0.126075	2.31355	3.827625	0.07875	0.3777167	0.0848583	0.5379583	3.1893538	0.0955167	0.1535833	0.295325	3.3559417	0.07295

* *P* value <0.05.

**Table 5:** Resume of variance analysis for metabolites identified in roots. (SV) Source of Variation; (DF) Degrees of freedom. (DAF) Days after flooding.

S.V	D.F	**Carbohidrates**				**Organic acids**					**Amino acids**				**Isoflavones**		
		Sucrose	Fructose	Glucose	Trehalose	Acetate	Citrate	Succinate	Fumarate	Malate	Alanine	Gaba	Choline	Pinitol	Malonyl	Daidzin	Daidzein
**Mean square (MS) 2 DAF**																	
G	1	2.074509*	0.03017	0.012832	0.000016	0.00081*	0.090446	0.004784*	0.000375	0.94905	0.036587*	0.008889*	0.002217*	0.160545*	768*	990.08*	10034.08*
WC	1	5.326935*	0.006538	0.010215	0.00121*	0.22897*	4.029611*	0.423151*	0.00839	0.063817	0.213227*	0.077281*	0.002152*	0.008933	85.3333	6.7500	2610.7500
G*WC	1	0.315382*	0.039159	0.004536	0.000012	0.00878*	0.02905	0.000791	0.000587	0.562424	0.032054*	0.007651*	0.000118	0.030281	341.333*	36.7500	3502.0833
Error	8	0.010462	0.017854	0.005249	0.000014	0.000125	0.059832	0.000321	0.000038	0.052094	0.000073	0.000084	0.000465	0.00567	25.5000	45.9167	1702.7500
CV		6.09	9.38	9.74	7.2	2.89	12.88	3.5	9.41	6.85	4.45	6.19	2.62	7.64	12.42	33.19	19.02
Mean		1.6808	1.4248583	0.7435325	0.0513917	0.3872	1.8997	0.5117333	0.0655083	3.3315583	0.1919333	0.14815	0.2904917	0.9851167	40.6667	20.4167	216.9167

**Mean square (MS) 7 DAF**																	
G	1	0.501925*	0.370797*	0.007213	0.000275*	0.006585*	0.596525*	0.168626*	0.002174*	5.215459*	0.009369*	0.000074	0.005096*	0.060265	0.04308*	0.0186*	0.0000
WC	1	12.76584*	0.697068*	0.160916*	0.000365*	0.784948*	3.036007*	1.906663*	0.006098*	1.235914*	0.152934*	0.104963*	0.005432*	0.008122	0.07192*	0.0180	0.0039
G*WC	1	0.692256*	0.039975	0.014255	3.33E-07	0.002423*	0.994925*	0.057727*	0.001784*	3.269704*	0.011072*	0.00012	0.000022	0.01068	0.027937*	0.0098	0.0121
ERROR	8	0.30115	0.009245	0.002955	0.000018	0.002309	0.014116	0.001201	0.000014	0.085525	0.000095	0.000074	0.000095	0.049444	0.0001	0.0035	0.0066
CV		9.85	7.27	6.92	14.82	3.36	5.8	5.74	6.69	8.73	5.73	5.26	3.3	8.13	7.12	100.88	40.69
MEAN		1.7622167	1.32345	0.7859167	0.0282333	0.5055917	2.049417	0.6037583	0.055275	3.3488583	0.1700583	0.163375	0.2958917	0.96735	0.1278	0.0583	0.1998

**Mean square (MS) 12 DAF**																	
G	1	0.46409*	0.000766	0.017055	9.08E-07	0.002491*	0.025098	0.010878*	0.000216*	0.518918*	0.000079	0.007071*	0.000137	0.06771*	0.00517*	0.018644*	0.0010
WC	1	2.904876*	0.003913	0.117889*	0.000072	0.248803*	8.176082*	0.33137*	0.001793*	0.370516*	0.007767*	0.022197*	0.008705*	0.570201*	0.001141*	0.00696*	0.1198*
G*WC	1	1.157973*	0.025604	0.001875	0.000031	0.026725*	0.001434	0.018229*	0.000068*	0.1565*	0.001178*	0.001494*	0.00012	0.06777*	0.002914*	0.005941*	0.0075
Error	8	0.007663	0.007938	0.006681	0.000038	0.000169	0.026335	0.000327	0.000056	0.019594	0.000035	0.000042	0.000073	0.002028	0.0001	0.0003	0.0097
CV		6.89	7.86	11.36	23.58	3.9	11.89	5.5	8.54	6.86	6.03	3.97	3.01	4.65	9.35	27.23	37.83
Mean		1.2709258	1.133225	0.71955	0.0260917	0.333475	1.3649667	0.328575	0.0310917	2.0397	0.009302	0.162525	0.28395	0.9680833	0.0816	0.0673	0.2608

* *P* value <0.05.
